# Completion of Second Volume

**Published:** 1863-07

**Authors:** 


### EDITORIAL DEPARTMENT.

### COMPLETION OF SECOND VOLUME.

This number makes us just two years old. Born at a time of the greatest commercial depression and distress, and at the beginning of the most fearful civil war ever known upon the globe; yet we have been spared to the age of two years. A very large proportion of those born under the most favorable circumstances never arrive at this period; but are cut off untimely and die in what is called the infancy of existence. The causes of this mortality with the young are exceedingly numerous, and no educated physician but is familiar with the influences which are most active. We shall not therefore go into details of the dangers we have escaped, but simply say that we are ourselves astonished, that we have attained to such venerable age, considering the great mortality attending this period of life; and the unparalleled fatality which has befallen both the old and young, the high and low, rich and poor, of our community. We could with pride and pleasure, relate some of the influences by which we have been preserved, and tell how that the vital principle of our existence being centered in the very heart of us, and not in any outside or foreign body, was one of the reasons why we still live, and shall continue to live, when those who depend upon collateral support, will have been long dead.

The Buffalo Medical and Surgical Journal is for the profession, and for nothing else. It has no object, other than the "greatest good of the greatest number" of physicians, and on this account mainly, we hope it will continue to make its monthly report. Perhaps it will be well to speak of the signal blessings it has enjoyed, for which it is deeply thankful. It has received constantly increasing "rations," nourishing and stimulating its growth. It has received many pleasant compliments from its subscribers, and from others who are capable of appreciating its qualities, which for one of its age, is somewhat important. When it is older and more fully established this will not be so necessary for it, as during its period of young life. It has received also new books, and new editions of books, pamphlets, addresses, speeches, &c., &c., besides the polite literature, magazines, reviews, quarterlies, &c. It has enjoyed unsurpassed opportunities, mainly through the favor of publishers and authors of medical books, editors and publishers of medical journals and of other popular journals and magazines.

Stimulated by our success, and encouraged by our rapidly increasing subscription list, we have concluded upon an enlargement of our Journal, which will hereafter contain forty pages, and be published at the price of $2.00 per annum. We do not double the number of our pages at first, yet we hope to, in a short time, when the expense of publication will enable us to do so. Meanwhile this increase will enable us to furnish our subscribers with a greatly improved Journal. We do not desire to make great promises for the future, but will let the Journal speak for itself.

We would say in this connection, that the subscription price of our Journal has been heretofore much too low for the present advanced prices of publication, and we have waited with some impatience for the completion of this volume, and favorable oportunity to make this change. We do this with some hesitation, and yet with the fullest confidence that our patrons and friends will at once recognize the necessity, and cheerfully aid us in our earnest endeavors to furnish an acceptable Journal, at the least possible expense.

We most cordially invite contributions for our pages, and hope we shall be supplied with the "choicest copy." We are under the deepest obligations to our contributors for the generous manner we have been served, and trust similar favors may be continued.

It will be our object to make the Buffalo Medical and Surgical Journal complete in all its arrangements, supplying the practitioner with whatever is new and interesting in the various departments of medical knowledge. At the same time it will be most thoroughly practical; conducted by one who has little time to devote to the examination of theories and speculations, but is engaged in active professional duty, it cannot be expected that the speculations of the theorist, independent of the knowledge of careful observation would be given space, which may more profitably be devoted to the record of fixed facts. We hope our friends will aid us, in making one of the most useful and valuable medical publications, and that they will also make effort to extend its influence, since there are yet a great many physicians who are "*without a knowledge of its value;*" indeed we fear that there are some, who are ignorant of the value of this or any other periodical medical publication; still this is not the direction in which we most desire to have our Journal extended.

There is something quite remarkable about this matter of subscribers for medical journals. In our own City there are three or four physicians who have never invited us to send them the Journal; and yet they are not greatly overburdened with other or better medical publications; they are not more familiar with the medical literature of their own or other countries; in a word, they as much require the knowledge they might obtain as others of the profession. When for any purpose it is desirable to learn who are the most capable, living, growing, intelligent men in the profession in the towns and cities which legitimately make Buffalo a medical centre, we have only to turn to our subscription list and we find their names; and this is much more true of Buffalo than of other towns or cities. We think it is no compliment to a physician here, if his name is not upon our subscription list, though it may in some instances be a compliment to the Journal. We have no reason to complain of the profession in Buffalo; we are rather proud of it, and proud that we receive from it an almost unexceptionable support. The profession of Buffalo and of Western New York, indeed the profession generally, have placed us under a deep sense of lasting obligation.

